# Graphene-oxide loading on natural zeolite particles for enhancement of adsorption properties†

**DOI:** 10.1039/c9ra00572b

**Published:** 2020-01-28

**Authors:** M. R. Silva, A. Lecus, M. Gajdardziska-Josifovska, M. Schofield, M. Virnoche, J. Chang, J. Chen, D. Garman

**Affiliations:** Water Technology Accelerator (WaTA), University of Wisconsin-Milwaukee 247 W. Freshwater Way Milwaukee Wisconsin 53204 USA msilva@uwm.edu; Department of Physics, University of Wisconsin-Milwaukee Mitchell Hall 251 Wisconsin 53201 USA; Department of Mechanical Engineering, University of Wisconsin-Milwaukee 3200 North Cramer Street Milwaukee Wisconsin 53211 USA; Centre for Infrastructure Engineering, Western Sydney University Room XB.3.34, Building XB, 56 Second Avenue Kingswood 2747 NSW Australia

## Abstract

Multiple methods of grafting graphene oxide (GO) nanosheets to natural clinoptilolite-rich zeolite particles were developed in our laboratory. In this study, we have systematically characterized the GO coated particles prepared by various methods to select the most promising method for further research efforts. This study revealed that the most promising coating method was the clean-acid-treated zeolite particles followed by deposition of GO nanosheets onto the zeolite surface and mild thermal treatment of the particles. GO and its synergistic interaction in zeolite was attributed to electrostatic interactions, hydrophobic interactions and hydrogen bonds. Hydrophobic interactions are enhanced both due to dealumination of zeolite caused by the cleaning method followed by acid treatment and due to partial thermal deoxygenation of GO. This method provided a ten times larger surface area (from 10.55 m^2^ g^−1^ to 117.96 m^2^ g^−1^) and three times smaller pore diameter (from 81.91 Å to 30.68 Å), providing great particles for a variety of applications as adsorbents or catalysts.

## Abstract 2

Multiple methods of grafting graphene oxide (GO) nanosheets to natural clinoptilolite-rich zeolite particles were developed in our laboratory.

## Graphical abstract

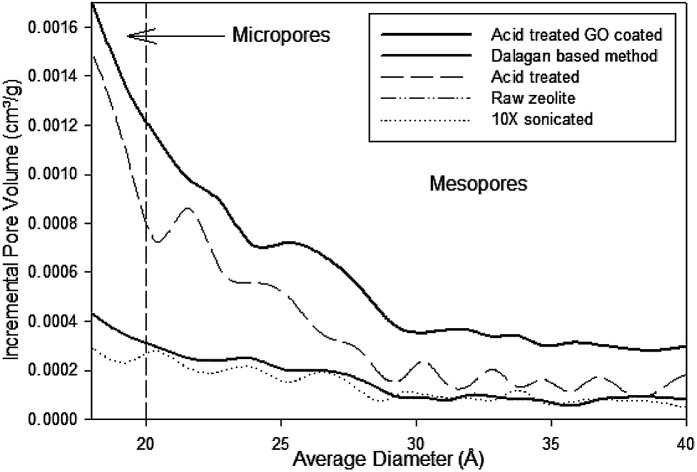

## A Introduction

Zeolites have been investigated for over two and a half centuries and is a well-established technology used in multiple processes and industries, ranging from construction materials and detergent builders, to catalysts and separation agents. As evidence of a vibrant activity in zeolite research, besides the 50 naturally occurring zeolites that have been identified, there are over 150 synthetic zeolites have been prepared and characterized;^1^ and 176 types of zeolite frameworks.^2^ Although environmental applications of zeolites are small compared with applications of their catalytic properties, some research and implementation has been performed in radioactive waste, water treatment and wastewater treatment.^1^

The flexible tectonic structure and ability to be chemically “tailored” to specific target species continues to stimulate their development. There is more need for further research leading to environmental applications. Zeolites are naturally occurring crystalline aluminosilicates, compositionally similar to clay minerals, but differing in their well-defined three-dimensional nano- and micro-porous structure. Aluminum, silicon, and oxygen are arranged in a regular structure of [SiO_4_]^−^ and [AlO_4_]^−^ tetrahedral units that form a framework with small pores (also called tunnels, channels, or cavities) of about 0.1–2 nm diameter running through the material. Generally they contain silicon, aluminum and oxygen in their framework and cations, water and/or other molecules within their pores.^3^ In addition, zeolites are economically attractive material, having low-cost (0.03–0.12 US$ per kg), with wide geographic distribution and large size of deposits.^4^

Functionalization of the surface of a zeolite changes the material in ways determined by the functional group. Properties that can be varied include surface charge, hydrophobicity, molecular binding and reactivity. Functionalization can be used to take advantage of known interactions. Some examples are amine binding to copper, sulfur binding to lead or gold and electrostatic interactions between positively charged surfaces and negatively charged anions, or between long chain *n*-alkylsilanes and hydrophobic organic contaminants. The different properties of the functional groups are used for reactions, and the reactions can be localized and controlled by processes such as bifunctionalization-putting two different functional groups in two different places on the zeolite, or by making larger structures with the zeolites and functionalizing various parts.

Besides, zeolites, other adsorbents have been used for removal of cations, such as heavy metals, with different mechanisms. Biosorbents are a versatile technology, have a variety of functional sites and it can be applied as hybrid technology and immobilization technology.^5^ Dry biofilms from biotrickling filters, for example, has been used to remove heavy metals from synthetic wastewater.^6,7^ Intracellular uptake and storage through active cationic transport systems and surface binding are the probable mechanisms.^7^ Biochars have also been used for removal of heavy metals from water. Biochar modified by surfactants can enable the simultaneous removal of heavy metals and surfactants from water^8^ and has also been used in composting and soil remediation^9^ Ligno-cellulosic material has been used for removal heavy metals from water through a sorption mechanism of combined chemical process involving surface chelation and ion exchange.^10^

In the case of natural zeolites, the sorption mechanisms of cations to natural zeolites is adsorption^11^ and it also has an ion-exchange nature,^11,12^ which can be enhanced by different methods. We selected graphene oxide (GO) to enhance physical properties of zeolite such as surface area and porosity GO is covalently decorated with oxygen-containing functional groups—either on the basal plane or at the edges—so that it contains a mixture of sp^2^- and sp^3^-hybridized carbon atoms. Due to the presence of oxygen functionalities, GO can easily disperse in organic solvents, water, and different matrices.

The aim of the current study is to develop and evaluate multiple methods of graphene-oxide loading on natural zeolite particles, characterizing properties of these materials and selecting the most promising method for further improvement. The expected benefits of this technology are to be able to use a natural material as a substrate, therefore enabling the use in large scale, while obtaining a stable particle, with high adsorption capacity and thermal stability and with competitive adsorption properties. There is intense interest in graphene and zeolite in fields such as physics, chemistry, and materials science, among others. Characterization of the engineered zeolite particles is essential to explore future applications.

## B Experimental

### Materials and chemicals

Australian natural clinoptilolite zeolite (diameter: 0.7–1 mm, chemical composition: 68.26% SiO_2_, 12.99% Al_2_O_3_, 4.11% K_2_O, 2.09% CaO, 1.37% Fe_2_O_3_, 0.83% MgO, 0.64% Na_2_O, 0.23% TiO_2_) was provided by Zeolite Australia Ltd. Graphene oxide was produced from natural graphite powder (SP-1, Bay Carbon, MI) using the modified Hummers' method.^13^ The GO suspension with a concentration of 2.5 g mL^−1^ was prepared by dispersing the prepared GO powder into deionised (DI) water with the assistance of ultrasonication for 10 min (Branson M1800 Ultrasonic Cleaner, 40 kHz). Other chemicals used in this study were sulfuric acid reagent grade 95–98% (Sigma Aldrich, USA) and ethyl-alcohol anhydrous (Electron Microscopy Sciences, USA).

### Materials characterization

The surface area (*S*_BET_), pore size and total pore volume distribution were determined by N_2_ adsorption isotherm with relationship between N_2_ adsorbed value at standard conditions (*V*) and the partial pressure (*p*/*p*_0_) under −196 °C (ASAP 2020, Micromeritics Inst. Corp.). Results for pore size distribution (d*V*/d*D*) were obtained from using Barrett–Joyner–Halenda (BJH) method with Faas correction. Before analysis, samples were pre-treated by degassing at 150 °C for 2 h, for removing any adsorbed species. Surface area calculations were made using the Brunauer–Emmett–Teller (BET) equation. The morphology and composition of the zeolite particles were characterized using a Hitachi S-4800 field emission scanning electron microscope (FESEM). Samples were mounted with conductive silver paste (EMSdiasum, 12686-15) on SEM stubs and viewed at an accelerating voltage of 5 kV. Transmission electron microscopy (TEM) and selected area electron diffraction (SAED) studies were performed in a Hitachi H9000NAR high resolution (HR) TEM using 300 keV electrons. Samples were ground with an agate mortar and pestle and supported on Cu TEM grids covered with amorphous holey carbon films. Thermogravimetric analysis (TGA) was carried out using a TA Instruments SDT 2960 Simultaneous DSC-TGA thermoanalyzer with a heating rate of 5 °C min^−1^ in air atmosphere. Raman spectra was obtained using a Horiba Scientific XploRA PLUS Raman microscope at a 532 nm wavelength spectra Zeta potential measurements were performed in a zeta potential ZETASIZER Nano Series-Nano ZS (Malvern Instruments Ltd, UK). X-Ray diffraction data was obtained using a Bruker ASX D8 Discover A25 device with a Cu tube at 1.5418 Å. Samples were placed in a zero-diffraction sample plate and measured from 10 to 60 2-theta.

### Synthesis of graphene oxide (GO)

GO was synthesized from natural graphite powder by a modified Hummers' method: 0.50 g of graphite powder was added to a mixture of 0.250 g NaNO_3_ and 13.0 mL of 98% H_2_SO_4_, followed by stirring for 1 hour in an ice bath at 0 °C. Subsequently, 1.50 g of KMnO_4_ was slowly added to the suspension while maintaining its temperature below 20 °C. The mixture was stirred at room temperature under reflux condition overnight. Then, 35.0 mL water was slowly added with vigorous stirring. The reaction temperature rapidly increased to 98 °C. Then, 6.0 mL of 30% H_2_O_2_, 20.0 mL of 10% HCl and 20.0 mL water were added to the mixture until its color was changed to brilliant yellow. The mixture was filtered and washed with water until the pH was close to 7. Finally, the obtained GO was collected and dried at 60 °C.

### Preparation of clean zeolite (zeolite 10X)

Zeolite and DI water (ratio 1 : 12.5 (w/v)) were placed in a beaker and sonicated (Fisherbrand, FB 11201, 37 kHz) 10 times for 15 minutes with three rinses in between each cycle. After sonication, the zeolite was boiled in a microwave at low power (zeolite : DI water ratio 1 : 10 (w/v)) for 30 minutes and the water was discarded and replaced after every boiling cycle. The zeolite was then dried in the oven for 24 h at 100 °C.

### Acid treatment of natural zeolite particles

Zeolite 10X was mixed with concentrated H_2_SO_4_ (zeolite : acid 2 : 1 ratio – w/v). The components were mixed in a boiling flask and refluxed for 12 hours between 80–90 °C. The zeolite was washed after 12 hours with absolute ethanol, centrifuged, and dried at 100 °C for 24 hours.

### Methods of coating of zeolite particles with graphene oxide

We developed and performed several functionalization methods as follows.

#### Modified Dalagan's method

Two grams of zeolite 10X was mixed with 1 mL concentrated H_2_SO_4_ and 25 mL of graphene oxide (2.5 mg mL^−1^). The components were mixed in a boiling flask and refluxed for 24 h at 100 °C. The zeolite was washed after 24 h with absolute ethanol, centrifuged, and dried at 100 °C. This method was based on a published method.^14^

#### Dip coating method

A dip coating device was designed out of a 15 mL test tube, a rod, and a plankton bucket net (0.008 mm × 0.012 mm). The rod was inserted in the cap of the test tube so the test tube could be dipped. A 50 mL test tube had a hole drilled in the center of its cap to fit the rod. The GO solution was placed in the 50 mL test tube and the zeolite was placed in the dip coater, the dip coater was placed in the 50 mL test tube and was vortex mixed for 24 hours upright. After 24 hours the dip coater was pulled out at a rate of 30 seconds to ensure a thin layer of coating. Once the zeolite was out of solution it was dried for 24 hours at 100 °C.

#### Spray coating method

Spray coating was performed with a Central Pneumatic air eraser kit. GO was prepared by diluting with DI water (water : GO 3 : 1 w/v ratio). The inside of the upper part of a filtering apparatus was coated lightly with the GO solution and then 1 g of zeolite was added to the filtering apparatus. The remainder of the solution was sprayed onto the zeolite and the samples were dried for 24 hours.

#### Plasma etch method

One gram of zeolite 10X was placed in a glass Petri dish and 1 mL of GO was distributed on the top. Sample was placed in the plasma etcher (Zepto, Diener Electronics), where vacuum was initially generated under 20 Pa and then increased to 80 Pa. Once the desired vacuum was reached, sample was submitted to plasma etching for 27 s at 50% power using oxygen and 10 psi (68.9 kPa) output. After the sample was plasma etched, sample was stirred immediately and mixed well and then dried at 100 °C for 24 h.

#### Spin coating method

Spin coating was done by placing zeolite in the lid of a small Petri dish with GO (zeolite : GO 1 : 1 ratio w/v). The mixture was ramped at 10 rpm, was dwelled for 15 seconds up until the sample reached 500 rpm. The samples were dried for 24 hours.

#### Sorption experiments

Adsorption experiments were conducted for brief assessment of comparison of performance among different zeolite particles and desorption experiments were performed for brief assessment of desorption of metal ions under given conditions for insight of regeneration of the particles. Full adsorption studies and modelling and full regeneration studies are beyond the scope of this study. Batch mode experiments on adsorption of cadmium on zeolite at initial concentration of 5 mg L^−1^ and ionic strength of 10^−3^ NaNO_3_ was conducted at a room temperature of 21 °C. Zeolite (0.5 g) was added to 100 mL cadmium solution to provide adsorbent loading rate of 5 g L^−1^ contained in glass flasks were sealed and agitated at 120 rpm for 24 h in an orbital shaker. Zeolite particles saturated in adsorption experiments were used for batch mode experiments on desorption of cadmium from each of the zeolites. Each of the zeolites (0.5 g) was added to 100 mL 1 M HCL contained in glass flasks and were sealed and agitated at 120 rpm for 24 h in an orbital shaker. In both adsorption/desorption experiments, the suspensions were filtered at 0.45 μm syringe filter and cadmium concentrations were measured in triplicates using Atomic Absorption Spectrometer (Thermo Scientific iCE™ 3300 AAS) and the average values were taken for data analysis. The amount of cadmium adsorption at equilibrium *q*_e_ (mg g^−1^), was calculated using eqn (1)1
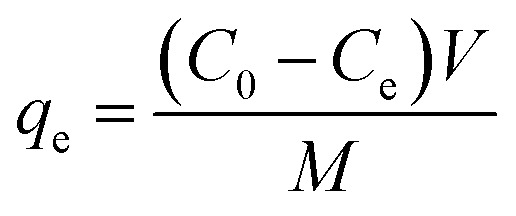
where *C*_0_ = initial concentration of heavy metal (mg L^−1^); *C*_e_ = equilibrium concentration of the heavy metal (mg L^−1^); *V* = volume of the solution (L) and *M* = mass of adsorbent (g).

Percent desorption efficiency (% DE) was calculated using eqn (2):2
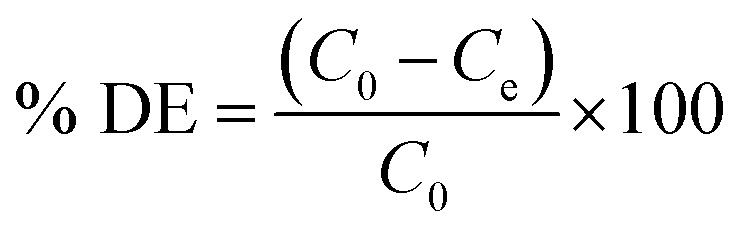


## C Results and discussion

### Structural characterization of natural zeolite, acid treated zeolite and GO–zeolite

The raw Australian zeolite has pore diameter ∼ 80 Å and when cleaned (10X sonicated zeolite), the pore diameter increases slightly. Fig. ESI-1† depicts pore size of zeolite particles under different cleaning and coating treatments. Multiple methods of coating zeolite with graphene oxide were evaluated and they represent the first phase of development of this hybrid zeolite graphene oxide materials, which have some limitations as lack of homogeneity in the amount of loading of GO and distribution. For all methods, our goal was to depart from the same concentration of graphene oxide and establish comparison between methods. Although the nominal loading of GO is 2.5 mg g^−1^, it is known that some methods have significant loss during the process such as spray coating. This type of observations among different methods of fabrication was part of the process of comparing methods. While attainment of uniformity and thin coating is important, we do not expect any of the methods presented in this paper to be able to provide uniform loaded graphene oxide as presented. The GO depositions on the zeolite surfaces are non-homogenous as Islands of GO deposits can be found on the surface of the coated particles in the SEM images, where sheet-like structures not typical for zeolite are observed (Fig. ESI-4†). We have developed further methods,^15^ but they are not subject of this paper. Cleaned particles were treated with a Dalagan based method, showing a very high standard deviation in pore size, as the method presents many operational variabilities. Spin coated, spray coated, and dip coated methods present average pore diameter slightly larger than 80 Å. The plasma etched and GO spin coated particle presents significantly increase in pore size, just over 100 Å. When clean zeolite particles are exposed to acid treatment, pore diameter decreases significantly (∼30 Å) as the pores get unclogged and shrink, holding this property after spin coated with GO.

**Fig. 1:**
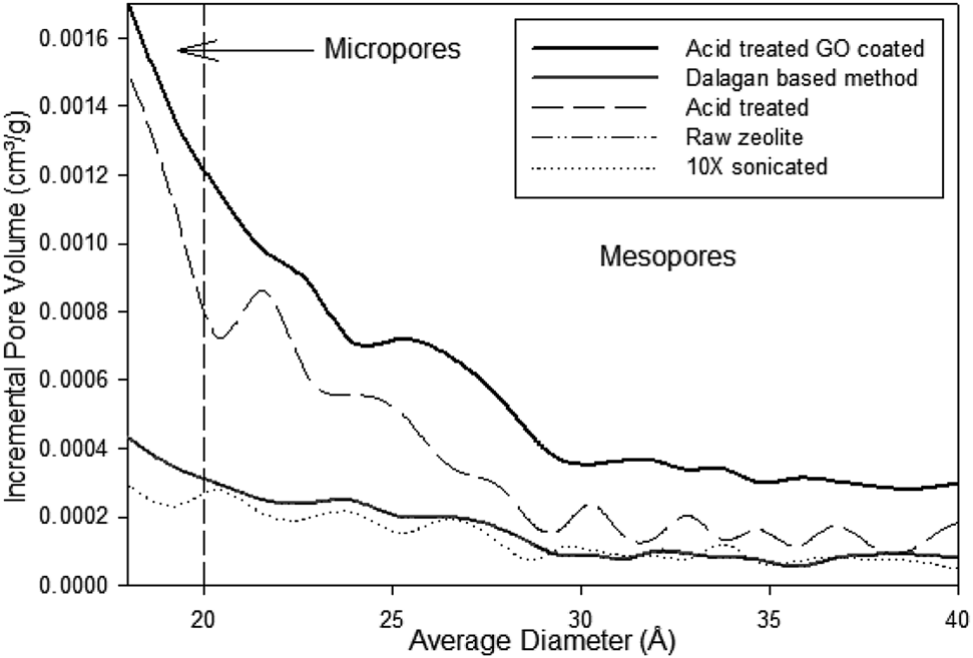
Pore size distribution of zeolite particles under different treatment and coatings using BJH method and Faas correction.

According to the IUPAC^16^ the classification of pore sizes within the range of 20 Å to 500 Å is defined as mesoporous and the range from 7 Å to 20 Å is supermicroporous. Pore size distributions for the zeolite particles are shown in Fig. 1. Where the data obtained was generated by graphical differentiation (d*V*/d*D*) with respect to diameter. All the samples show a continuous distribution extending well into the micro region. Particles receiving acid treatment have predominant area under the curves to the left of *D* = 20 Å, denoting increasing significant micropore influence, especially the acid treated GO coated zeolite.

**Fig. 2:**
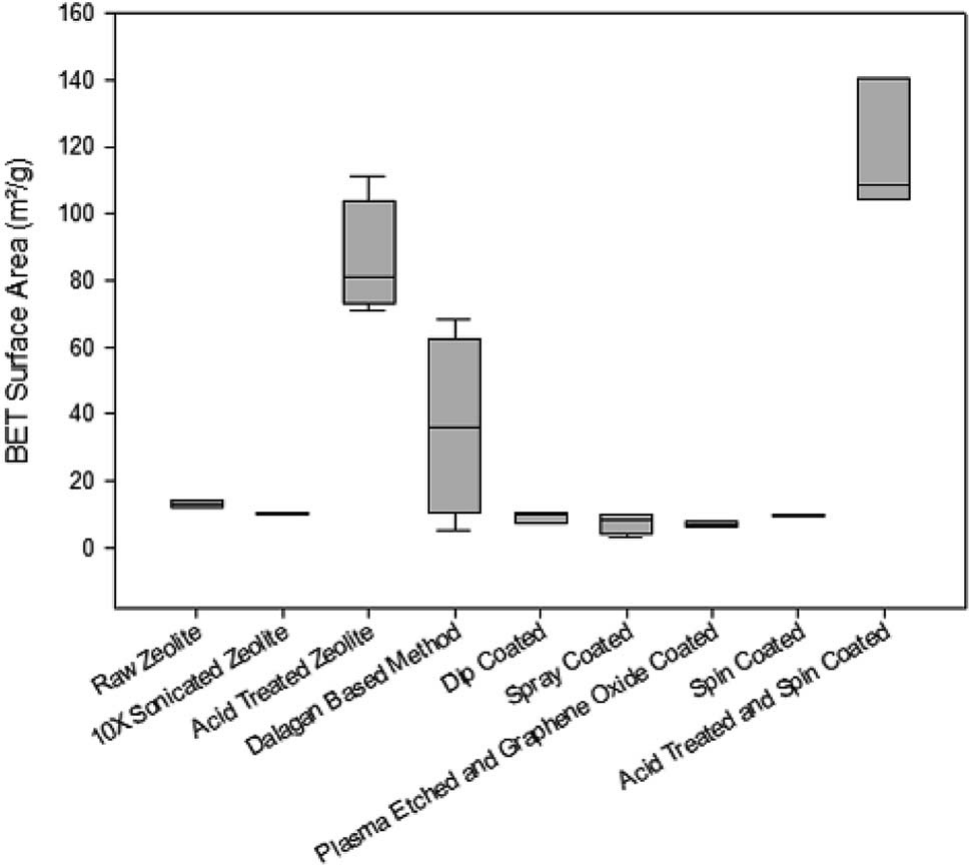
Surface area of zeolite particles under different cleaning and coating treatments.

Evaluation of the BET surface area of the zeolites (Fig. 2) show that the cleaning and most of GO coating methods maintain the same surface area of the raw material (∼10 m^2^ g^−1^). Surface area increases when particles are exposed to acid treatment. Dalagan based method provides mean surface area of ∼40 m^2^ g^−1^, with substantial variability. When clean particles are exposed to 12 h acid treatment, surface area increases eight times, reaching 80 m^2^ g^−1^ and when GO coated it exceeds 100 m^2^ g^−1^.

Most particles show pore volume at around 0.02 cm^3^ g^−1^ (Fig. ESI-2†). Pore volume increase when particles are exposed to acid treatment. Particles fabricated though Dalagan based method have pore volume around 0.04 cm^3^ g^−1^ with great variability. Clean zeolite particles that went through 12 hour acid treatment show 0.06 cm^3^ g^−1^ and just over 0.08 cm^3^ g^−1^ when GO coated. This suggests that dealumination due to acid treatment interferes with pore architecture.

**Fig. 3:**
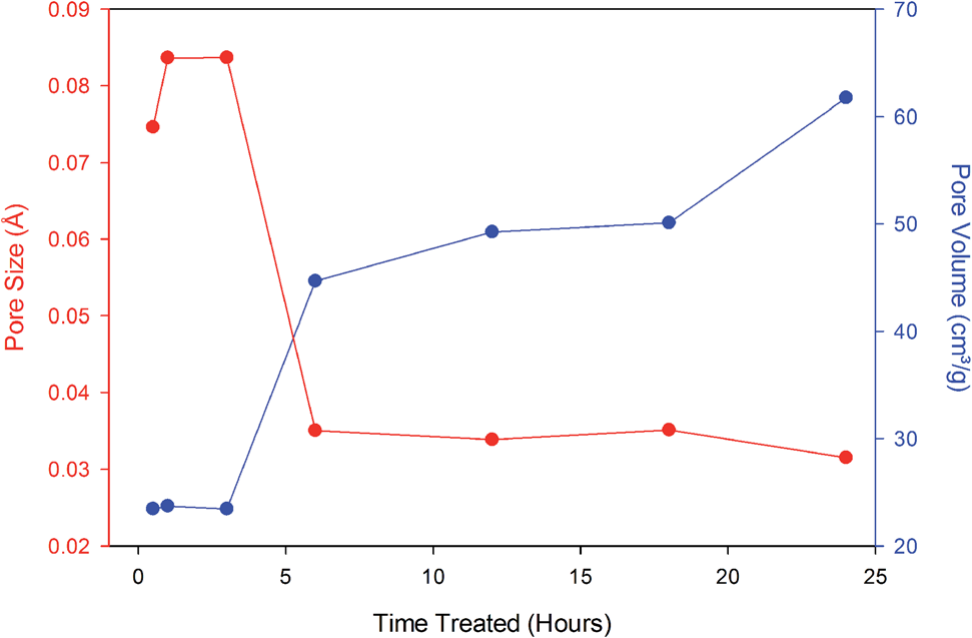
Single point pore volume and average pore size of zeolite particles under acid treatment over time.

To verify this hypothesis, we further examined the effects of acid reflux on the clean zeolite particles (Fig. 3). The single point pore volume was obtained by taking the sum of all the pore volumes within a specified range. The data shows the pore volume increases with increased exposure to the acid treatment at temperature of 85 °C. The most substantial change in pore volume occurs at 6 hours with a pore volume of 0.054 cm^3^ g^−1^. As time of acid treatment progresses, dealumination is known to take place as the volume of the pores increase while the pore diameter decreases up to 6 h when it stabilizes around 30 Å. Dealumination is a method of chemical and structural modification of zeolites, where decationation takes place. Very common dealumination process consists of the treatment of samples in inorganic or organic acid.^17^ Under action of the introduced protons, ≡SiO–(Al(−))≡ bonds became hydrolyzed and changed firstly into more stable ≡Si–O–H units plus H–O–Al≡ split structure. Framework aluminum tends to reduce pore volume. It is also known that steaming reduces pore volume, but not necessarily the pore dimensions.^18,19^ As the zeolite particles progress on the treatment process, they increase concentration of silica and reduce concentration of alumina (dealumination). Alumina-rich zeolites are attracted to polar molecules such as water, while silica-rich zeolites work better with nonpolar molecules.

**Fig. 4:**
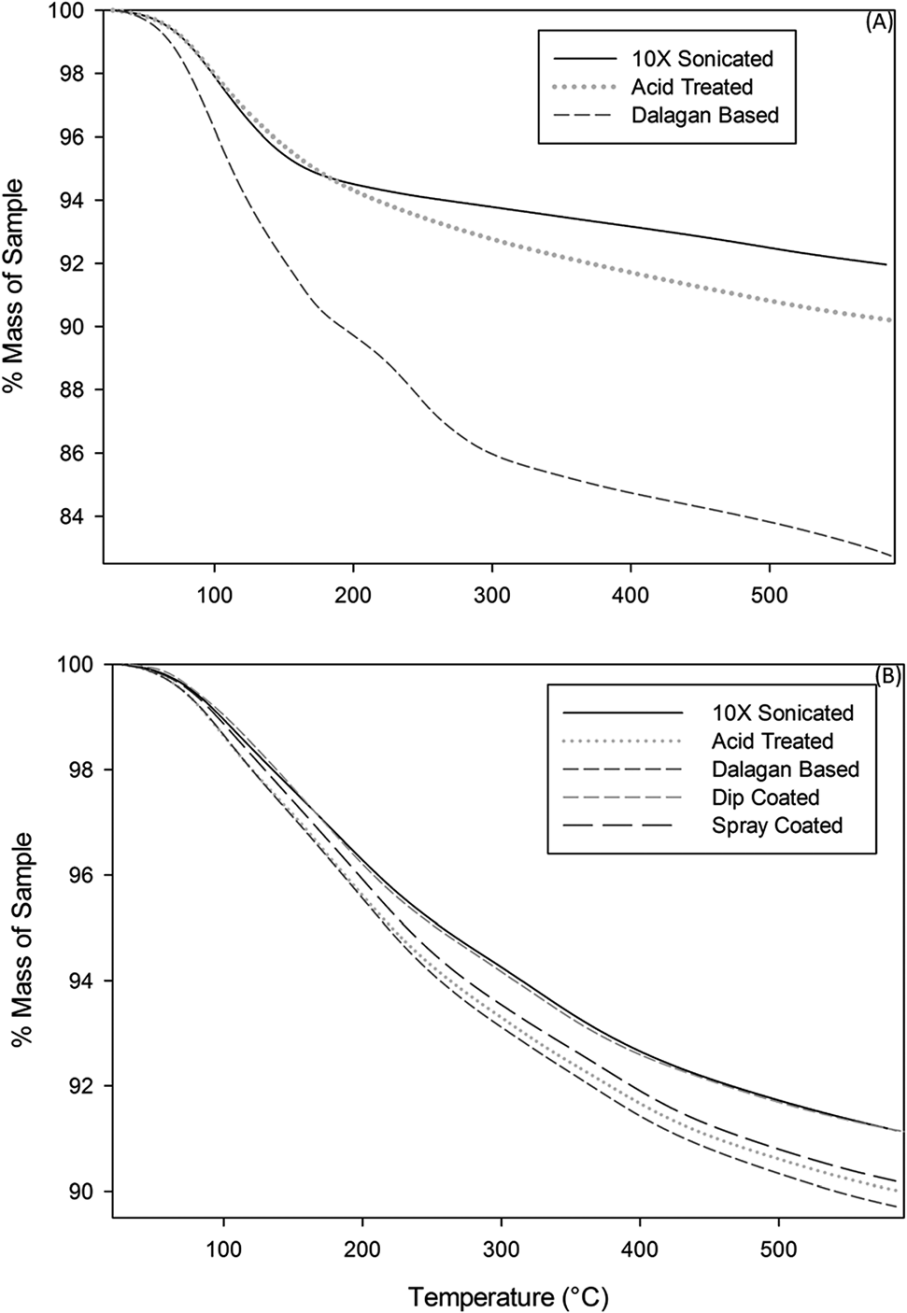
Thermogravimetric curves of zeolite particles exposed to (A) physical methods and (B) chemical methods.

Comparative TGA curves of the zeolites are presented in Fig. 4A and B. Because the zeolites coated with GO in this study may be used in processes that might require elevated temperatures or might need heat treatment during the regeneration process, it was deemed important to understand their thermal stability limits. From the thermographs, it is apparent that moisture loss from both zeolites treated with physical and chemical methods started around 50 °C and continued up to approximately 600 °C for physical methods and until about 400 °C for Dalagan based method and only about 200 °C for acid treated. Comparison of the percentage weight loss of zeolite exposed to physical methods showed approximately 10% percentage weight loss of moisture for all samples, whereas the zeolite treated with Dalagan based had a total loss of about 14% and the acid treated and further GO coated particle had a total loss of approximately 6%. All zeolite samples investigated show a sudden slope change at low temperatures (<100 °C), indicating the presence of water molecules which are weakly bonded to the surface.^20,21^ The weight loss occurring above 200 °C can be associated with structural water due to hydration complexes formed with exchangeable cations.^22^ The water loss above 400 °C can be associated with dihydroxylation, formed when exchangeable cations polarize water molecules, leading to release of more water from the zeolite cavities. Removal of a portion of the tetrahedrally coordinated aluminum from the zeolite framework has been shown to increase its thermal stability, which is often directly proportional to Si : Al ratio.^22^ The reason for this increase in stability might be attributable to the formation of new Si–O–Si bonds. The four hydroxyls in the product of reaction are each bonded to silicon; on heating, these four groups would be expected to condense to yield water and Si–O–Si bonds.^17^

**Fig. 5:**
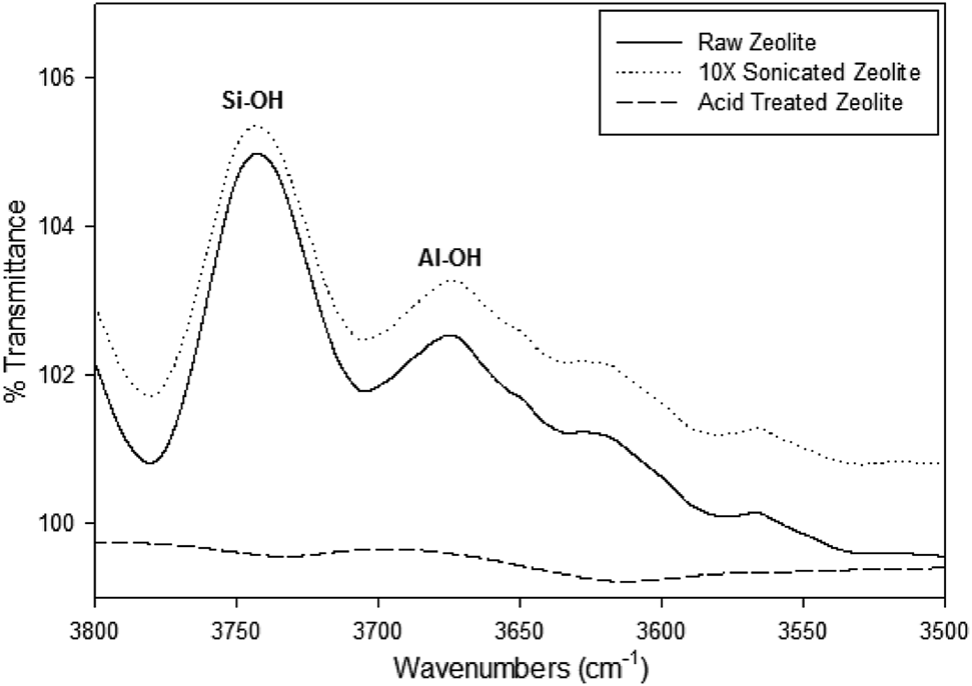
FTIR spectra of O–H stretching vibrations in the region located between 3500 and 3800 cm^−1^ of the zeolite samples at different stages of fabrication.

Dealumination of the zeolite samples can be detected by FTIR in the spectral range of 3800 to 3500 cm^−1^ showing the Si–OH and Al–OH bands, which are associated with the delamination of the zeolite.^23^Fig. 5 shows a small variation of the signal around 3746 cm^−1^ of O–H stretching vibrations of silanols after the acid treatment. This contrasts with the strong diminution of the signal at 3610 cm^−1^ which is consistent with the reduction of the number of Si–OH–Al groups and the precipitation of aluminum outside the zeolite framework, throughout the fabrication process (from raw zeolite to acid treated GO coated).

The peak 3720–3740 cm^−1^ and 3770 cm^−1^ are associated with terminal silanol groups (SiOHs) with Al in an octahedral-like environment, very likely terminal too, which also reduce throughout processing of the particles.^24,25^ Both of the peaks at 3742 cm^−1^ in the Si–OH region and a slight peak at 3670 cm^−1^ in the Al–OH region can be distinguished in the Raw and 10X sonicated zeolite samples, however these peaks become undetectable in the 12 hour acid treated zeolite sample. This opens the possibility that the increase of the hydrophobic character, that accompanies the partial exit of tetrahedrally coordinated aluminum out of the crystalline network, by effect of the hydrothermal treatment, causes the diminution of the bands at 3700–3642 cm^−1^.^26^

### Surface characterization, evaluation of GO coating, and mechanisms of attachment of GO to zeolite particles

**Fig. 6:**
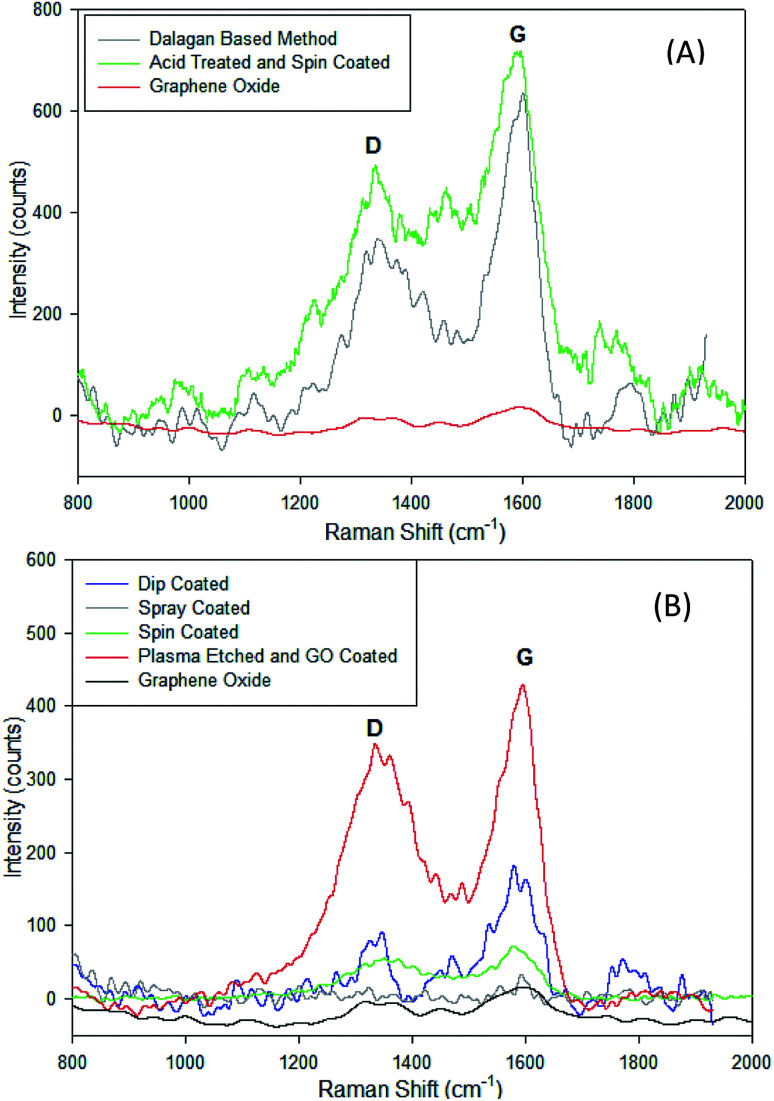
Comparison of Raman spectra of zeolite particles coated with GO with (A) chemical methods and (B) physical methods showing the D (∼1350 cm^−1^) and G (∼1585 cm^−1^) relative peak heights.

Presence of GO on the surface of zeolite can be demonstrated through Raman spectra. The Raman spectra observed from 800 to 2000 cm^−1^ shows the presence of graphene oxide at the G and D peaks (Fig. 6A and B). The G band (at ∼1585 cm^−1^) characterizes the sp^2^-hybridized carbon–carbon bonds in graphene and occurs due to carbon–carbon bonds stretching in graphene, while D peak (at ∼1350 cm^−1^) is the disorder-induced band at the edge of graphene sample. The D band appears when the laser hits the edges of the graphene sheets as a result of symmetry breaking and change in selection rules.^27^ Both the Dalagan based and the acid treated spin coated samples show a presence of graphene oxide deposits. In the physical methods of coating, each method except the spray coated zeolite show a clear presence of graphene oxide on the sample. Increasing ratios at these peaks indicate that reduction of the material is occurring (Fig. 6A and B). The acid treated and spin coated sample shows the greatest reduction of the graphene for the chemical-based method, and the plasma etched sample shows the greatest reduction among the physical coating methods.

**Fig. 7:**
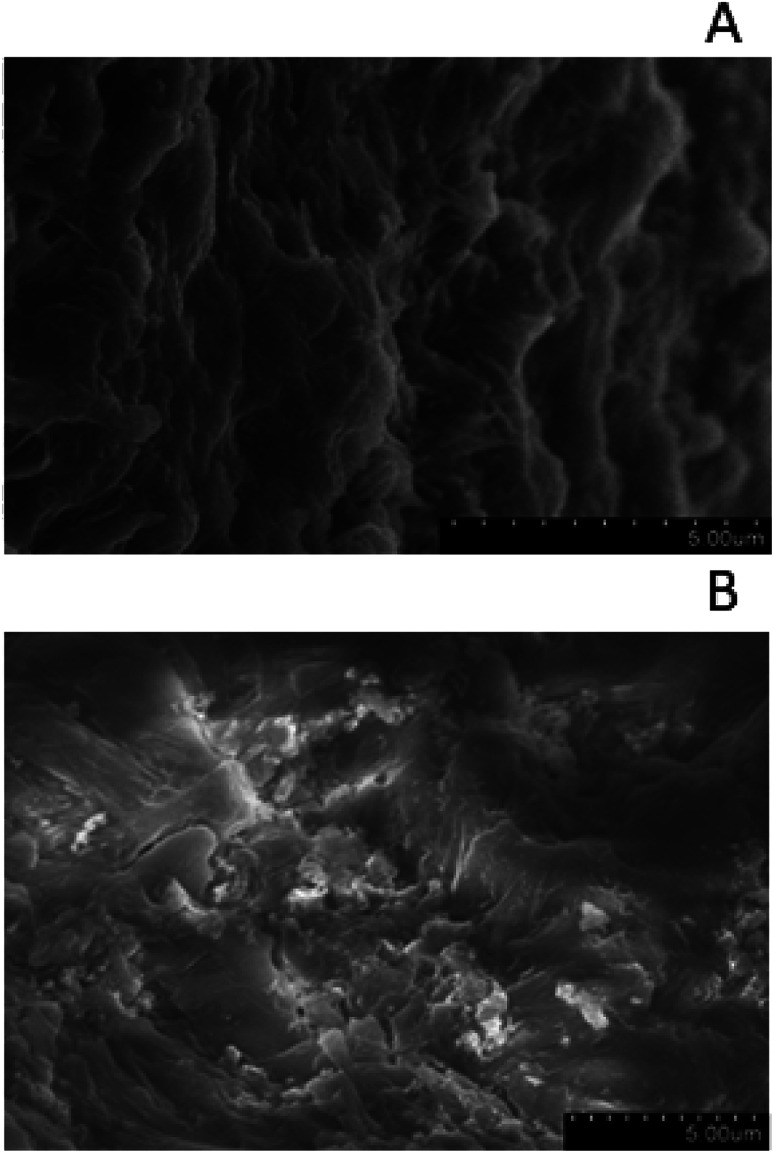
(A) SEM of clean zeolite (zeolite 10X) showing typical elongation features. (B) SEM of acid treated clean zeolite particles showing small zeolite grains.

FE-SEM images for representatives of clean zeolite (Fig. 7A) and acid treated zeolite (Fig. 7B). Clean zeolite has typically elongated features, which are also observed on the raw zeolite particles (image not shown), however several small particles, probably dust, cover the surface of these features. After prolonged acid treatment, elongated features are rare, and zeolites show outline sketch of smaller grains, supporting the previous analysis on the pore developments and structures. FE-SEM observations suggest that prolonged acid treatment form small zeolite grains. Graphene oxide was observed on GO zeolite particles. GO are transparent veil-like structures, containing highly-wrinkled graphitic layers caused by the distortion in the graphene layers due to the linkage of the residual oxygen after thermal reduction, while large nanosheet sizes are preserved (image not shown), as described elsewhere.^28^

**Fig. 8:**
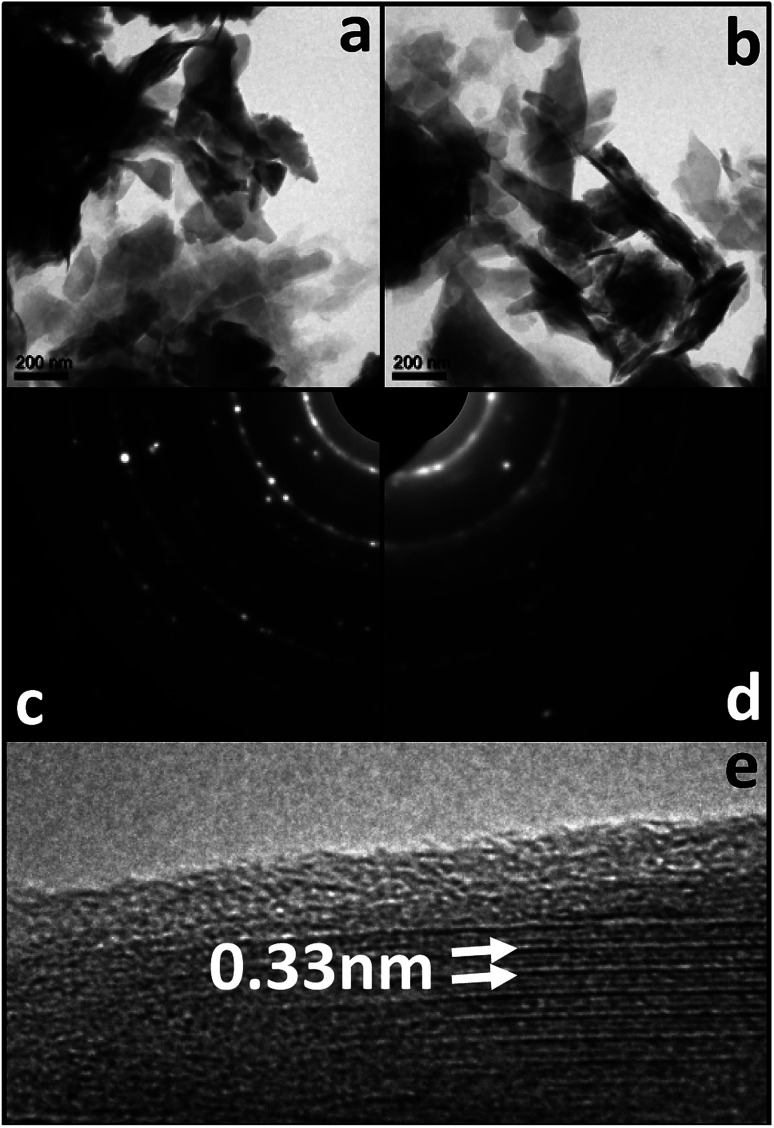
TEM images and respective SAED patterns from (a and c) acid treated zeolite and (b and d) acid treated and GO spin coated zeolite. HRTEM from acid treated and GO spin coated zeolite (e) shows lattice planes with spacing of 0.33 nm covered with an amorphous looking ∼1 nm surface layer.

Bright field TEM images in Fig. 8 show very similar morphologies for acid treated zeolite (Fig. 8a) and spin coated zeolite (Fig. 8b). These amplitude contrasts TEM images are recorded at relatively low magnification of 7000× due to radiation sensitivity of all uncoated zeolite samples that prevented phase contrast HRTEM lattice imaging. Spin coating with GO increases the stability of the zeolite and allows HRTEM imaging (Fig. 8e).

Lattice fringes with 0.33 nm periodicity are visible in domains with width of a few nanometers, and the surface is coated with ∼1 nm thick disordered (amorphous-like) layer. The polycrystalline ring SAED patterns from the two sample types show sharp rings for the acid treated zeolite (Fig. 8c) which become broadened and less intense for the spin coated zeolite (Fig. 8e). In order to obtain further insight on the role of GO and its synergistic interaction in zeolite, we characterized the zeolite particles by XRD (Fig. ESI-5†). No significant distinctive peak was observed between zeolites with or without GO, indicating no significant change in the crystalline lattice detected by XRD.

The zeta potential values of GO dispersed in water are negative at pH range from 4 to 10 (Fig. ESI-3†), which has also been reported by Shubin Yang *et al.*^29^ The negative charges originated from the ionization of the carboxylic acid and phenolic hydroxy groups located on the GO. The values of zeta potential for clean zeolite are negative at pH range from 2 to 12.

The first mechanism of attachment of GO to zeolite is electrostatic interactions. The mechanism of coating GO sheet on the zeolites surface is mainly the interactions of the interlayered hydrogen bonds for facilitating GO sheet adherence to the zeolites surface – electrostatic interactions between GO–GO particles and GO–zeolite particles.

Deposition of particles on to surfaces is very often controlled by the zeta potential of particles and collectors.^30^ Interaction between GO–GO and GO–zeolite particles can be evaluated through measurement of zeta potentials across a range of pH of the suspensions of the particles, zeta potential values of GO and zeolite particles at various pH values were assessed (Fig. ESI-3†). Electrostatic interactions has been cited as mechanism of removal of heavy metals by biochar when surface-sorbed by an anionic surfactant, giving biochar negative charges, which improved the electrostatic attraction between biochar and Cr(iii), however it enhanced repulsion between biochar and Cr(iv).^8^ Graphene oxide contains epoxide and hydroxyl functional groups, and ions such as Na can be adsorbed vertically onto the oxygen atoms of the graphene oxide-epoxide (GO-epoxide). The adsorption energy for this case was reported to be approximately twice the value for the adsorption of Na onto the pristine graphene, probably due to the doping effect of the oxygen in the epoxide, enhancing the adsorption energy.^31^

In this study, both GO and zeolite are negatively charged for all the pH values with the exception of GO at pH 2 (2 ≤ pH ≤ 12), suggesting that GO–zeolite and GO–GO interactions are repulsive. Similar findings for GO–zeolite and GO–GO interactions have been reported in the literature by several other investigators.^32^ Zeta potentials of both GO and zeolite decrease with increasing pH, suggesting that the suspended particles become more stable with increasing pH. Stability of suspended particles is expected to increase with increasing absolute zeta potential values.^33^ The commonly used threshold for absolute zeta potential value for stable colloidal suspensions is considered to be >30 mV.^34^ Therefore, particle suspensions with pH > 5.5 are desired, because both GO and zeolite particles are stable.

The second possible mechanism are hydrophilic/hydrophobic interactions. GO particles are mostly hydrophilic and are also known for their amphiphilic properties as well. Natural zeolite particles are also amphiphilic particles. The zeolite hydrophobicity and hydrophilicity strongly depend on the aluminum content, which affect adsorption of polar and apolar molecules into the zeolite.^35^ The hydrophobicity is enhanced with dealumination.^36^ This makes the GO coated zeolite a versatile engineered material for targeting a broad range of compounds from water.

The third possible mechanism of attachment is hydrogen bond network formed between oxygen functionality on GO and water,^37^ present in the graphene oxide solution or in the intramolecular water molecule in the zeolite framework. All the water molecules in zeolite are bonded to extra framework cations and every hydrogen atom is H-bonded to framework oxygens.^38^

**Fig. 9:**
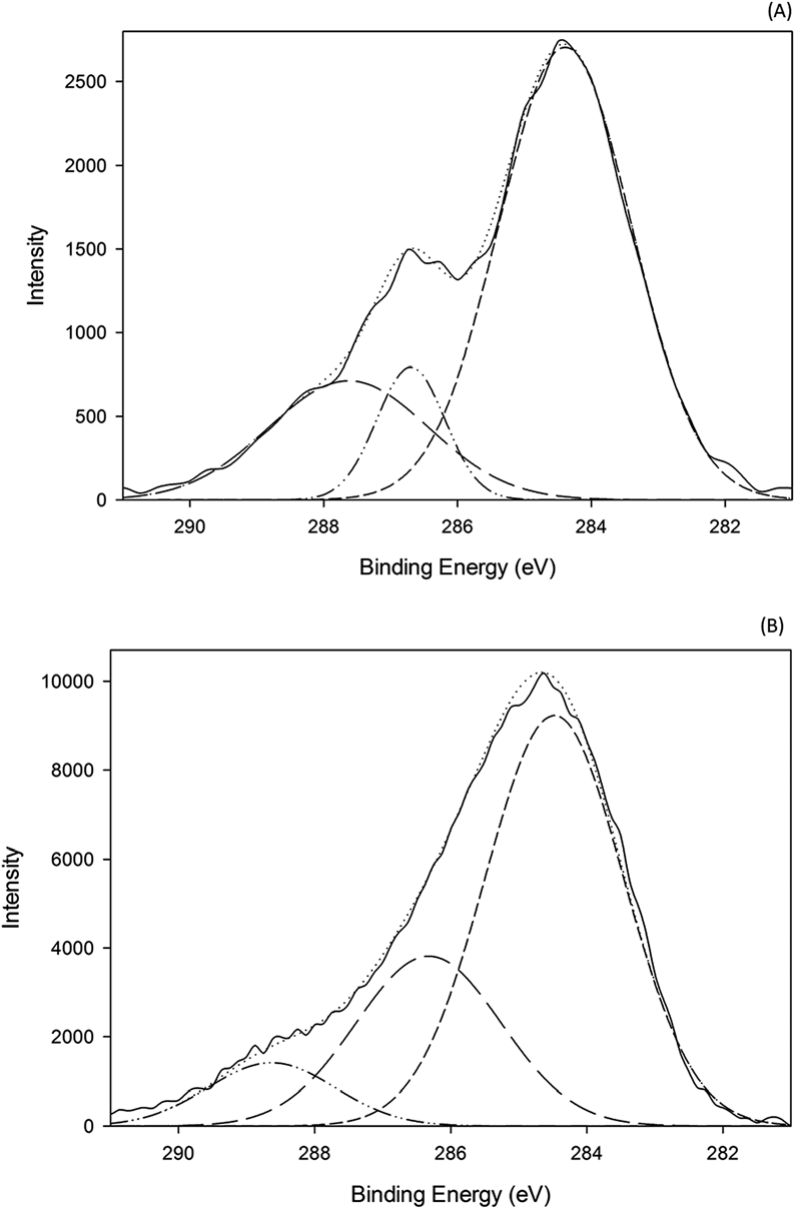
(A) XPS data for graphene oxide and (B) acid treated GO coated zeolite.

X-ray photoelectron spectrometry (XPS) was performed to confirm the predominant mechanism. Results (Fig. 9) show one large peak at 284 eV in the C1s region for both the GO sample and the acid treated GO coated sample. The GO sample shows a very intense and wide peak at 287 eV which is reduced in the acid treated GO coated sample. However, the peak at 286 eV in the acid treated GO coated is much more intense than in the GO. This peak corresponds to –C–N– bonds, which is associated with hydrophobic interactions. It would be highly unlikely that hydrogen bonding is the dominating mechanism of attachment because the samples are dried, in which case these interactions would be for the most part eliminated. For the case of the acid treated GO coated zeolite, hydrophobic interactions seem to be the dominating mechanism of attachment, however this does not mean it is the only mechanism of attachment. It is likely that hydrogen bonding has a greater impact when the zeolite is initially coated, however the dried material at the end of the process lacks their interactions. With acid treatment, dealumination leads to the increasing hydrophobic interactions as indicated in the XPS data.

### Sorption experiments of zeolites

Brief assessment of performance of all zeolite particles is detailed in Table ESI-1.† Adsorption performance was similar for removal of cadmium ions and varied from 71% to 78%. On the other hand, their desorption properties varied significantly among zeolites. Desorption of cadmium from most of the particles varied from 89% to 99% under the experimental conditions. Three of the zeolites, dalagan based method, acid treated zeolite and acid treated and spin coated did not have significant desorption of cadmium ions (from 0% to 19%, respectively) showing more stability under these conditions. Literature has reported vast list of methods of desorption and regeneration for zeolites that can be further explored for our particles, with vast variability in percent removal (from 30% to 100%) and desorption efficiency (from 24% to 99.5%), depending on the adsorbent, heavy metals, experimental conditions and technology applied.^39^ Further work is needed to optimize the method of recovery of heavy metals and regeneration of adsorbent. As per adsorption capacity, it has reported to vary from ∼6 mg g^−1^ (natural zeolite) to ∼1600 mg g^−1^ synthetic nanozeolite A for cadmium ions. Synthetic zeolites are expected to perform better as they are fabricated under controlled conditions, fine-tuned for better performance and homogeneous. The adsorption capacity of our zeolites varied from 661 mg g^−1^ to 720 mg g^−1^, which is a great performance for a naturally based material further improved with physical treatments (cleaning and acid treatment) and/or engineered with graphene oxide, allowing applications at large scale in water industry, which estimated cost is $4/kg, being competitive when compared to the market price of the granular activated carbon (GAC) of 5 to 15 $ per kg (Table ESI-2†).

From all the coating methods tested and presented in this study, the acid treated and GO spin coated zeolite particle appears to have better overall performance, in terms of larger surface area, smaller pore diameter and larger pore volume, the most thermally stable material, with clear indication of GO coating evidenced by Raman, SEM and TEM, while maintain good performance of adsorption capacity when compared with the other zeolites. It is expected good adsorption properties with cationic, anionic and non-ionic compounds and broad application of this material.

## D Conclusions

This work presents several proposed methods of coating natural zeolite particles with graphene oxide. Through extensive analysis, it is possible to show that zeolite particles that are cleaned and acid treated, followed by GO spin coating have better adsorption properties. These particles enhanced external surface area, smaller pore diameter, larger pore volume, improved thermal stability and stable at pH > 5.5. Raman spectroscopy and SEM and TEM imaging demonstrate the presence of graphene oxide in the zeolite surface. Potential mechanisms of attachment are electrostatic interactions, hydrophobic interactions and hydrogen bond interactions.

## Conflicts of interest

There are no conflicts to declare.

## Supplementary Material

RA-010-C9RA00572B-s001
