# Supplementary material for: Graphene-oxide loading on natural zeolite particles for enhancement of adsorption properties
Source: RSC Adv. 2020 Jan 28;10(8):4589–97. doi: 10.1039/c9ra00572b (PMC9049076; doi:10.1039/c9ra00572b)
Supplement: RA-010-C9RA00572B-s001 [file RA-010-C9RA00572B-s001.pdf]

Electronic Supplementary Material (ESI) for RSC Advances.
This journal is © The Royal Society of Chemistry 2020

Graphene-oxide loading on natural zeolite particles for enhancement of adsorption properties

M. R. Silva*[a], R. Lecus[a], M. Gajdardziska[b], M. Schofield[b], M. Virnoche [a], J. Chang [a,c] , J. Chen [a,c] and D. Garman[a]

Electronic supplementary information (ESI).

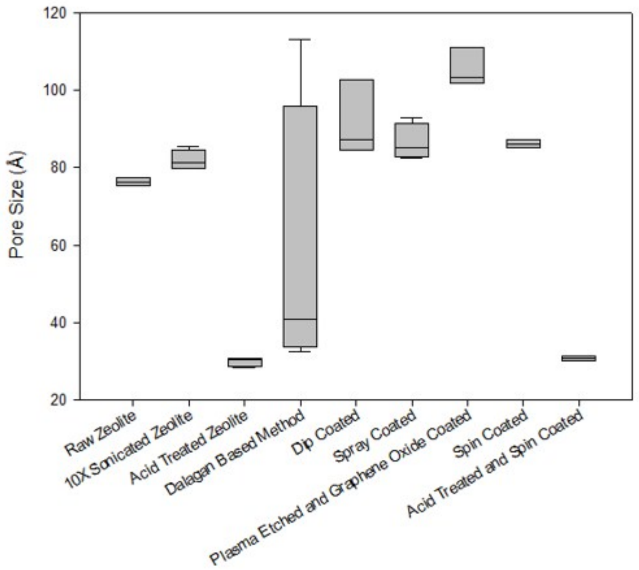

Figure ESI-1. Pore size of zeolite particles under different cleaning and coating treatments.

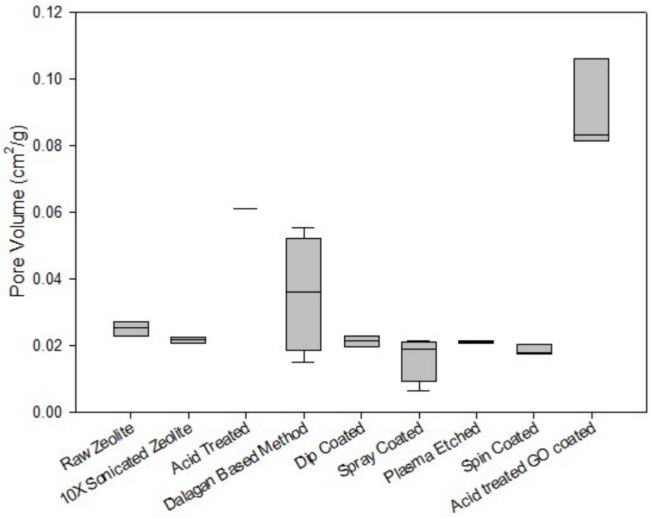

Figure ESI-2. Pore volume of zeolite particles under different cleaning and coating treatments.

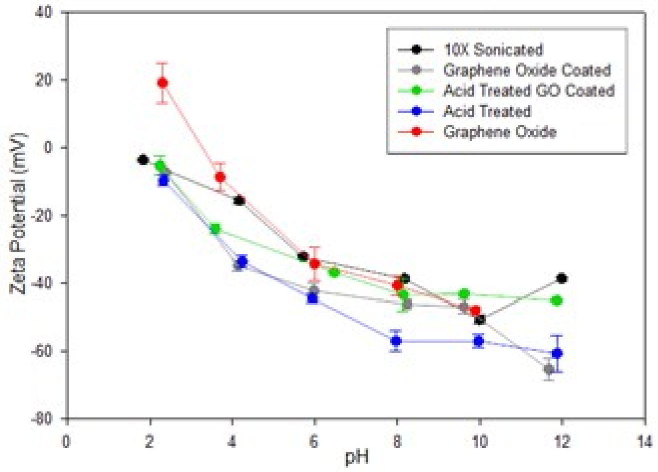

Figure ESI-3. Effect of solution pH on the zeta potential of zeolite particles and GO suspensions.

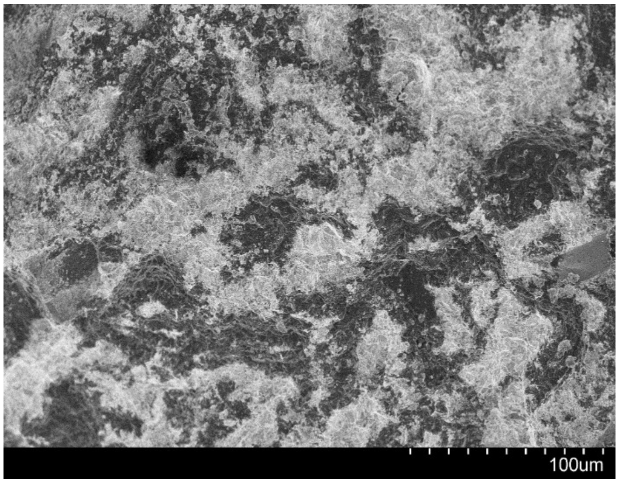

Figure ESI-4. SEM image of dip coated zeolite exemplifying observation of islands of GO deposits (darker spots), that can also be seen by unaided eye.

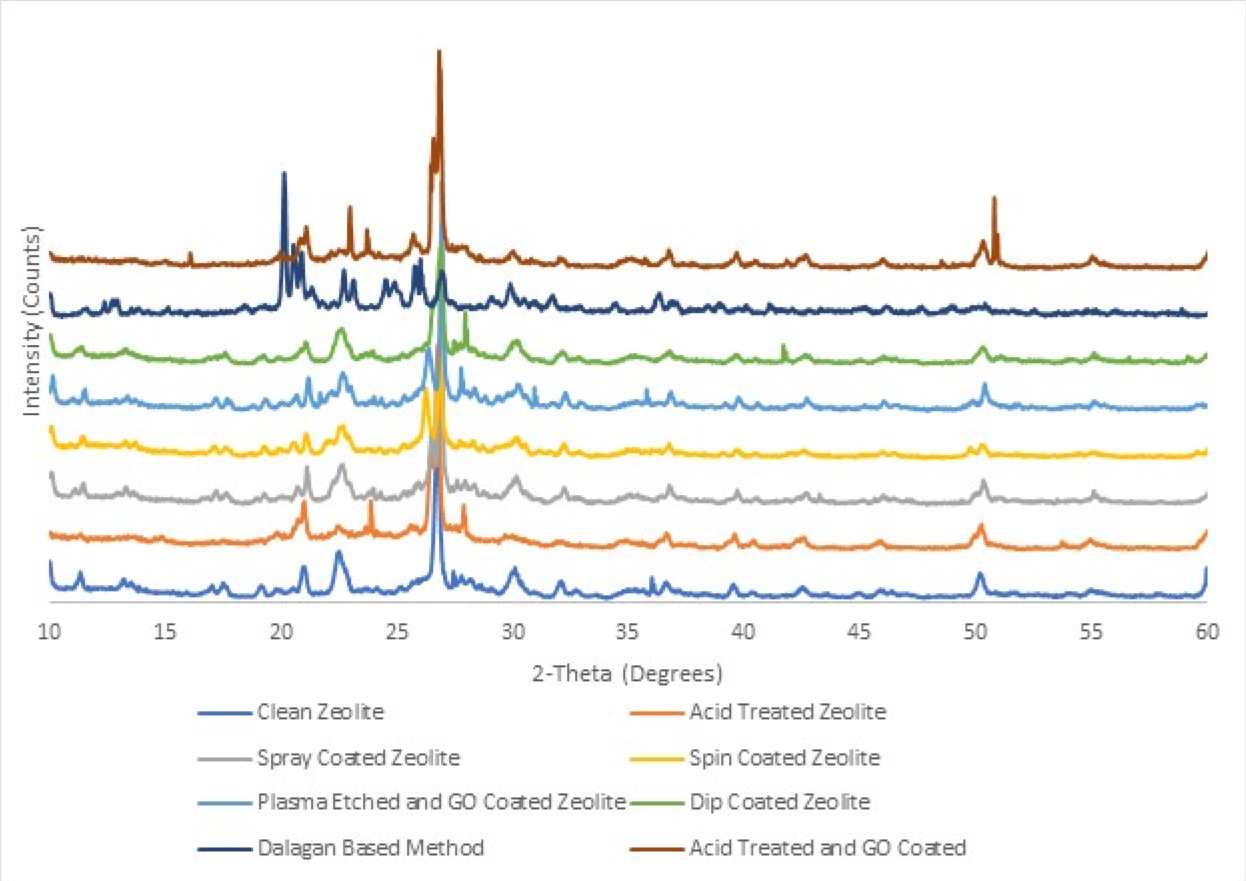

Figure ESI-5. XRD patterns of zeolites- clean, acid treated and coated with GO using different methods.

Table 1. Metal sorption and desorption percent by zeolite

Zeolites	Cd uptake mg/g	Adsorption % RE	Desorption % DE
Acid treated and spin coated	695	75	19
Clean zeolite	708	77	89
Spin coated	718	78	99
Dalagan based method	656	71	0
Dip coated	720	78	95
Acid treated zeolite	661	72	14
Plasma etched and GO coated	709	77	96
Spray coated	714	77	94

Table 2. Economic Analysis for Engineered zeolites applied in water treatment

Price of GO ($/g)	Price of AC ($/kg)	Price of natural zeolite ($/kg)	GO Theoretical loading (mg/g)	Our material prices (not including synthesis process) ($/kg)		
				Lowest (Industry scale)	Medium	Highest (lab scale)
Lowest price (China Shandong) 0.3~0.5	5~15	0.2~1	2.5	0.95~1.13	3.5~63.5	125~375
Medium price (China Jiang Su) 1~25						
Supply made in the U.S. 50~150						

*The GO theoretical loading is the amount of GO added in the coating process, which should be higher than practice. Also, the GO can be recycled in the industry process.

* All the final prices are including the production cost.

The calculation for the cost our materials:

Take Medium price of GO in the market for example: $1/g

For the theoretical loading is 2.5, price of GO zeolite =1*2.5+0.5= $3/kg

The costs of expenses are around $0.5~1/kg to produce the materials, so the final price is ~$4/kg
